# Supplementary material for: The cost of aging: Economic growth perspectives for Europe
Source: PLoS One. 2023 Jun 23;18(6):e0287207. doi: 10.1371/journal.pone.0287207 (PMC10289460; doi:10.1371/journal.pone.0287207)
Supplement: S2 Appendix — (DOCX) [file pone.0287207.s002.docx]

**S2 Appendix. Dfuller drift test**

| **Country** | **PGDP** | **EPOP** | **DEPOP** | **DDEPOP** |
| --- | --- | --- | --- | --- |
| Austria | -6.095*** | 0.9960 | -1.5830 | -3.712*** |
| Belgium | -6.493*** | 1.0130 | -1.4200 | -4.361*** |
| Denmark | -6.53*** | 1.5160 | -1.0990 | -4.22*** |
| Finland | -5.01*** | 5.3750 | -1.0230 | -3.754*** |
| France | -5.681*** | 4.7210 | -1.1660 | -3.231** |
| Greece | -4.948*** | 1.2270 | -2.3360 | -7.646*** |
| Italy | -5.821*** | 2.7320 | -1.7260 | -3.266** |
| Luxembourg | -5.61*** | -4.889*** |  |  |
| Netherlands | -5.411*** | 9.7330 | 0.1080 | -3.874*** |
| Norway | -4.161*** | -1.7130 | -0.6050 | -3.966*** |
| Portugal | -4.867*** | 10.2000 | -1.1740 | -4.13*** |
| Spain | -4.956*** | 1.9910 | -1.2970 | -4.104*** |
| Sweden | -5.783*** | -2.5370 | -1.0790 | -5.295*** |
| Turkey | -7.589*** | 6.4380 | 1.0790 | -2.642* |
| United Kingdom | -6.786*** | -0.1980 | -1.5500 | -5.055*** |

Notes: *, denotes significance at the 10% level, **, denotes significance at the 5% level,***, denotes significance at the 1% level.

Source: Authors’ illustrations based on STATA software
