# Supplementary material for: The cost of aging: Economic growth perspectives for Europe
Source: PLoS One. 2023 Jun 23;18(6):e0287207. doi: 10.1371/journal.pone.0287207 (PMC10289460; doi:10.1371/journal.pone.0287207)
Supplement: S4 Appendix — (DOCX) [file pone.0287207.s004.docx]

**S4 Appendix. Stability test**

| **Eigenvalue** | | **Modulus** |
| --- | --- | --- |
| **Real** | **Imaginary** |  |
| 0.7442642 | 0.3355485 | 0.816408 |
| 0.7718106 | 0 | 0.7718106 |
| -0.1453613 | -0.4483621 | 0.4713369 |
| -0.1453613 | 0.4483621 | 0.4713369 |
| -0.3738819 | 0 | 0.3738819 |

Source: Authors’ illustrations based on STATA software.
