# Supplementary material for: The cost of aging: Economic growth perspectives for Europe
Source: PLoS One. 2023 Jun 23;18(6):e0287207. doi: 10.1371/journal.pone.0287207 (PMC10289460; doi:10.1371/journal.pone.0287207)
Supplement: S5 Appendix — (DOCX) [file pone.0287207.s005.docx]

**S5 Appendix. Lag length criteria results**

|  | **Lag** | **P** | **FPE** | **AIC** | **HQIC** | **SBIC** |
| --- | --- | --- | --- | --- | --- | --- |
| **Austria** | 0 |  | 0.02127 | 1.82515 | 1.85445 | 1.90237 |
|  | 1 | 0.00000 | .016391* | 1.56441 | 1.6523* | 1.79606* |
|  | 2 | 0.97400 | 0.01913 | 1.71763 | 1.86411 | 2.10371 |
|  | 3 | 0.01800 | 0.01769 | 1.63715 | 1.84222 | 2.17767 |
|  | 4 | 0.41700 | 0.01932 | 1.72046 | 1.98412 | 2.41541 |
|  | 5 | 0.28200 | 0.02066 | 1.78057 | 2.10283 | 2.62996 |
|  | 6 | 0.00100 | 0.01670 | 1.55741* | 1.93826 | 2.56123 |
|  | 7 | 0.22100 | 0.01775 | 1.60405 | 2.04349 | 2.76230 |
|  | 8 | 0.33800 | 0.01943 | 1.67463 | 2.17266 | 2.98732 |
|  | 9 | 0.09000 | 0.01991 | 1.67382 | 2.23044 | 3.14095 |
|  | 10 | 0.19900 | 0.02144 | 1.71471 | 2.32993 | 3.33628 |
| **Belgium** | 0 |  | 0.01555 | 1.51183 | 1.54112 | 1.58904 |
|  | 1 | 0.00100 | .012271* | 1.27493* | 1.36282* | 1.50658* |
|  | 2 | 0.45100 | 0.01342 | 1.36312 | 1.50960 | 1.74920 |
|  | 3 | 0.23000 | 0.01412 | 1.41191 | 1.61699 | 1.95243 |
|  | 4 | 0.05100 | 0.01377 | 1.38216 | 1.64583 | 2.07712 |
|  | 5 | 0.61000 | 0.01546 | 1.49046 | 1.81272 | 2.33985 |
|  | 6 | 0.00400 | 0.01339 | 1.33656 | 1.71741 | 2.34039 |
|  | 7 | 0.57200 | 0.01507 | 1.44037 | 1.87981 | 2.59863 |
|  | 8 | 0.92000 | 0.01776 | 1.58457 | 2.08260 | 2.89726 |
|  | 9 | 0.01000 | 0.01633 | 1.47513 | 2.03175 | 2.94225 |
|  | 10 | 0.00200 | 0.01401 | 1.28917 | 1.90439 | 2.91073 |
| **Denmark** | 0 |  | 0.01009 | 1.07910 | 1.10839 | 1.15632 |
|  | 1 | 0.00100 | .008209* | .872933* | .960821* | 1.10458* |
|  | 2 | 0.74900 | 0.00930 | 0.99681 | 1.14329 | 1.38290 |
|  | 3 | 0.22500 | 0.00978 | 1.04436 | 1.24944 | 1.58488 |
|  | 4 | 0.76400 | 0.01114 | 1.16997 | 1.43364 | 1.86493 |
|  | 5 | 0.31200 | 0.01198 | 1.23588 | 1.55813 | 2.08526 |
|  | 6 | 0.03000 | 0.01146 | 1.18068 | 1.56153 | 2.18450 |
|  | 7 | 0.11200 | 0.01174 | 1.19082 | 1.63026 | 2.34907 |
|  | 8 | 0.15500 | 0.01231 | 1.21829 | 1.71633 | 2.53098 |
|  | 9 | 0.31900 | 0.01351 | 1.28557 | 1.84219 | 2.75269 |
|  | 10 | 0.96200 | 0.01623 | 1.43643 | 2.05165 | 3.05799 |
| **Finland** | 0 |  | 0.01714 | 1.60909 | 1.63839 | 1.68631 |
|  | 1 | 0.00000 | .009857* | 1.0559* | 1.14379* | 1.28755* |
|  | 2 | 0.44600 | 0.01077 | 1.14341 | 1.28989 | 1.52949 |
|  | 3 | 0.60700 | 0.01203 | 1.25135 | 1.45642 | 1.79187 |
|  | 4 | 0.06000 | 0.01183 | 1.23032 | 1.49399 | 1.92528 |
|  | 5 | 0.56900 | 0.01322 | 1.33378 | 1.65604 | 2.18317 |
|  | 6 | 0.28700 | 0.01420 | 1.39492 | 1.77577 | 2.39874 |
|  | 7 | 0.40900 | 0.01564 | 1.47699 | 1.91643 | 2.63525 |
|  | 8 | 0.17400 | 0.01649 | 1.51038 | 2.00841 | 2.82307 |
|  | 9 | 0.22500 | 0.01774 | 1.55798 | 2.11461 | 3.02511 |
|  | 10 | 0.51900 | 0.02020 | 1.65515 | 2.27037 | 3.27671 |
| **France** | 0 |  | 0.01866 | 1.69432 | 1.72362 | 1.77154 |
|  | 1 | 0.00000 | .009356* | 1.00368* | 1.09157* | 1.23533* |
|  | 2 | 0.34300 | 0.01006 | 1.07526 | 1.22174 | 1.46134 |
|  | 3 | 0.21700 | 0.01056 | 1.12088 | 1.32596 | 1.66140 |
|  | 4 | 0.13200 | 0.01081 | 1.13962 | 1.40329 | 1.83458 |
|  | 5 | 0.82800 | 0.01243 | 1.27241 | 1.59466 | 2.12179 |
|  | 6 | 0.19600 | 0.01307 | 1.31228 | 1.69313 | 2.31610 |
|  | 7 | 0.33100 | 0.01422 | 1.38172 | 1.82116 | 2.53998 |
|  | 8 | 0.11700 | 0.01468 | 1.39454 | 1.89257 | 2.70723 |
|  | 9 | 0.19400 | 0.01567 | 1.43388 | 1.99050 | 2.90100 |
|  | 10 | 0.54900 | 0.01791 | 1.53484 | 2.15006 | 3.15640 |
| **Greece** | 0 |  | .031753* | 2.22598* | 2.25528* | 2.3032* |
|  | 1 | 0.13200 | 0.03237 | 2.24503 | 2.33292 | 2.47668 |
|  | 2 | 0.60000 | 0.03607 | 2.35215 | 2.49864 | 2.73824 |
|  | 3 | 0.13600 | 0.03691 | 2.37254 | 2.57761 | 2.91306 |
|  | 4 | 0.70900 | 0.04178 | 2.49207 | 2.75573 | 3.18702 |
|  | 5 | 0.93900 | 0.04875 | 2.63906 | 2.96131 | 3.48845 |
|  | 6 | 0.82400 | 0.05623 | 2.77144 | 3.15229 | 3.77527 |
|  | 7 | 0.87700 | 0.06554 | 2.91008 | 3.34953 | 4.06834 |
|  | 8 | 0.46000 | 0.07308 | 2.99949 | 3.49752 | 4.31218 |
|  | 9 | 0.30800 | 0.08002 | 3.06469 | 3.62132 | 4.53182 |
|  | 10 | 0.86700 | 0.09487 | 3.20216 | 3.81738 | 4.82372 |
| **Italy** | 0 |  | 0.02523 | 1.99608 | 2.02538 | 2.07330 |
|  | 1 | 0.00000 | .015134* | 1.48464* | 1.57253* | 1.71629* |
|  | 2 | 0.36300 | 0.01633 | 1.55946 | 1.70594 | 1.94555 |
|  | 3 | 0.02100 | 0.01521 | 1.48605 | 1.69112 | 2.02657 |
|  | 4 | 0.12700 | 0.01554 | 1.50290 | 1.76657 | 2.19786 |
|  | 5 | 0.60900 | 0.01744 | 1.61105 | 1.93331 | 2.46044 |
|  | 6 | 0.21300 | 0.01842 | 1.65551 | 2.03636 | 2.65934 |
|  | 7 | 0.34200 | 0.02007 | 1.72679 | 2.16623 | 2.88505 |
|  | 8 | 0.65000 | 0.02291 | 1.83967 | 2.33771 | 3.15236 |
|  | 9 | 0.15400 | 0.02415 | 1.86654 | 2.42317 | 3.33367 |
|  | 10 | 0.77200 | 0.02831 | 1.99305 | 2.60827 | 3.61461 |
| **Luxembourg** | 0 |  | 2.08449 | 6.41027 | 6.43922 | 6.48603 |
|  | 1 | 0.00000 | 0.08135 | 3.16644 | 3.25329 | 3.39371 |
|  | 2 | 0.00000 | .028478* | 2.11585* | 2.2606* | 2.49464* |
|  | 3 | 0.36600 | 0.03069 | 2.18832 | 2.39097 | 2.71863 |
|  | 4 | 0.36200 | 0.03310 | 2.26013 | 2.52067 | 2.94195 |
|  | 5 | 0.30900 | 0.03547 | 2.32299 | 2.64143 | 3.15632 |
|  | 6 | 0.08100 | 0.03558 | 2.31688 | 2.69323 | 3.30174 |
|  | 7 | 0.62000 | 0.04004 | 2.42200 | 2.85624 | 3.55836 |
|  | 8 | 0.43700 | 0.04425 | 2.50480 | 2.99694 | 3.79268 |
|  | 9 | 0.27300 | 0.04785 | 2.56077 | 3.11081 | 4.00017 |
|  | 10 | 0.70200 | 0.05519 | 2.67484 | 3.28278 | 4.26576 |
| **Netherlands** | 0 |  | 0.00323 | -0.05937 | -0.03007 | 0.01785 |
|  | 1 | 0.00000 | .002421* | -.347971* | -.260083* | -.11632* |
|  | 2 | 0.95000 | 0.00281 | -0.19925 | -0.05277 | 0.18683 |
|  | 3 | 0.77900 | 0.00320 | -0.07204 | 0.13304 | 0.46849 |
|  | 4 | 0.79100 | 0.00366 | 0.05654 | 0.32021 | 0.75150 |
|  | 5 | 0.55400 | 0.00408 | 0.15812 | 0.48038 | 1.00751 |
|  | 6 | 0.04000 | 0.00395 | 0.11661 | 0.49746 | 1.12043 |
|  | 7 | 0.67800 | 0.00451 | 0.23260 | 0.67204 | 1.39085 |
|  | 8 | 0.60400 | 0.00512 | 0.34011 | 0.83815 | 1.65280 |
|  | 9 | 0.94400 | 0.00608 | 0.48794 | 1.04456 | 1.95506 |
|  | 10 | 0.90700 | 0.00725 | 0.63044 | 1.24566 | 2.25200 |
| **Norway** | 0 |  | 0.00455 | 0.28260 | 0.31190 | 0.35982 |
|  | 1 | 0.00000 | .002109* | -.486259* | -.398371* | -.254608* |
|  | 2 | 0.26100 | 0.00223 | -0.43059 | -0.28411 | -0.04450 |
|  | 3 | 0.95900 | 0.00260 | -0.28032 | -0.07525 | 0.26020 |
|  | 4 | 0.04100 | 0.00251 | -0.32069 | -0.05703 | 0.37426 |
|  | 5 | 0.97000 | 0.00294 | -0.16828 | 0.15398 | 0.68111 |
|  | 6 | 0.29100 | 0.00316 | -0.10632 | 0.27453 | 0.89750 |
|  | 7 | 0.48400 | 0.00352 | -0.01361 | 0.42583 | 1.14465 |
|  | 8 | 0.85800 | 0.00412 | 0.12268 | 0.62071 | 1.43537 |
|  | 9 | 0.39700 | 0.00457 | 0.20289 | 0.75951 | 1.67001 |
|  | 10 | 0.51600 | 0.00521 | 0.29966 | 0.91487 | 1.92122 |
| **Portugal** | 0 |  | 0.00913 | 0.97915 | 1.00844 | 1.05636 |
|  | 1 | 0.00100 | .007418* | .771646* | .859534* | 1.0033* |
|  | 2 | 0.57700 | 0.00824 | 0.87601 | 1.02249 | 1.26210 |
|  | 3 | 0.28700 | 0.00879 | 0.93726 | 1.14233 | 1.47778 |
|  | 4 | 0.06300 | 0.00866 | 0.91822 | 1.18188 | 1.61317 |
|  | 5 | 0.10800 | 0.00880 | 0.92675 | 1.24901 | 1.77614 |
|  | 6 | 0.67800 | 0.00998 | 1.04281 | 1.42366 | 2.04663 |
|  | 7 | 0.94700 | 0.01175 | 1.19101 | 1.63045 | 2.34927 |
|  | 8 | 0.23400 | 0.01259 | 1.24072 | 1.73876 | 2.55341 |
|  | 9 | 0.23800 | 0.01359 | 1.29136 | 1.84798 | 2.75848 |
|  | 10 | 0.06800 | 0.01383 | 1.27656 | 1.89178 | 2.89812 |
| **Spain** | 0 |  | 0.01544 | 1.50511 | 1.53441 | 1.58233 |
|  | 1 | 0.00100 | .012101* | 1.26098* | 1.34886* | 1.49263* |
|  | 2 | 0.60900 | 0.01350 | 1.36909 | 1.51557 | 1.75517 |
|  | 3 | 0.25000 | 0.01427 | 1.42250 | 1.62758 | 1.96302 |
|  | 4 | 0.82100 | 0.01636 | 1.55450 | 1.81817 | 2.24946 |
|  | 5 | 0.33100 | 0.01766 | 1.62383 | 1.94609 | 2.47322 |
|  | 6 | 0.70500 | 0.02010 | 1.74284 | 2.12369 | 2.74667 |
|  | 7 | 0.82000 | 0.02327 | 1.87470 | 2.31414 | 3.03296 |
|  | 8 | 0.30200 | 0.02530 | 1.93873 | 2.43677 | 3.25143 |
|  | 9 | 0.19000 | 0.02697 | 1.97710 | 2.53373 | 3.44423 |
|  | 10 | 0.00000 | 0.02128 | 1.70741 | 2.32263 | 3.32897 |
| **Sweden** | 0 |  | 0.00602 | 0.56367 | 0.59297 | .640891* |
|  | 1 | 0.00600 | 0.00530 | 0.43467 | .522557* | 0.66632 |
|  | 2 | 0.28700 | 0.00564 | 0.49585 | 0.64233 | 0.88193 |
|  | 3 | 0.40000 | 0.00613 | 0.57662 | 0.78169 | 1.11714 |
|  | 4 | 0.00300 | .005238* | 0.41545 | 0.67912 | 1.11041 |
|  | 5 | 0.14600 | 0.00541 | 0.43978 | 0.76204 | 1.28917 |
|  | 6 | 0.04300 | 0.00526 | .401931* | 0.78278 | 1.40575 |
|  | 7 | 0.54300 | 0.00590 | 0.50215 | 0.94160 | 1.66041 |
|  | 8 | 0.26300 | 0.00636 | 0.55838 | 1.05642 | 1.87107 |
|  | 9 | 0.14400 | 0.00668 | 0.58176 | 1.13839 | 2.04889 |
|  | 10 | 0.43100 | 0.00752 | 0.66713 | 1.28235 | 2.28869 |
| **Turkey** | 0 |  | 0.00589 | 0.54111 | 0.57041 | 0.61833 |
|  | 1 | 0.00000 | .003116* | -.095719* | -.00783* | .135933* |
|  | 2 | 0.84800 | 0.00357 | 0.03944 | 0.18592 | 0.42553 |
|  | 3 | 0.29000 | 0.00381 | 0.10121 | 0.30628 | 0.64173 |
|  | 4 | 0.25600 | 0.00404 | 0.15581 | 0.41947 | 0.85076 |
|  | 5 | 0.11700 | 0.00412 | 0.16836 | 0.49061 | 1.01774 |
|  | 6 | 0.17400 | 0.00431 | 0.20185 | 0.58270 | 1.20567 |
|  | 7 | 0.78200 | 0.00496 | 0.32942 | 0.76886 | 1.48768 |
|  | 8 | 0.33700 | 0.00543 | 0.39984 | 0.89787 | 1.71253 |
|  | 9 | 0.72600 | 0.00629 | 0.52118 | 1.07780 | 1.98830 |
|  | 10 | 0.04600 | 0.00628 | 0.48660 | 1.10182 | 2.10816 |
| **United Kingdom** | 0 |  | 0.01136 | 1.19833 | 1.22763 | 1.27555* |
|  | 1 | 0.00400 | 0.00977 | 1.04746 | 1.13535* | 1.27911 |
|  | 2 | 0.33800 | 0.01050 | 1.11811 | 1.26459 | 1.50420 |
|  | 3 | 0.55300 | 0.01165 | 1.21953 | 1.42460 | 1.76005 |
|  | 4 | 0.00500 | 0.01013 | 1.07498 | 1.33865 | 1.76994 |
|  | 5 | 0.17500 | 0.01055 | 1.10886 | 1.43111 | 1.95825 |
|  | 6 | 0.00000 | .007738* | .788088* | 1.16894 | 1.79191 |
|  | 7 | 0.10300 | 0.00790 | 0.79432 | 1.23376 | 1.95257 |
|  | 8 | 0.14300 | 0.00824 | 0.81744 | 1.31548 | 2.13014 |
|  | 9 | 0.22500 | 0.00887 | 0.86485 | 1.42147 | 2.33197 |
|  | 10 | 0.04300 | 0.00883 | 0.82726 | 1.44248 | 2.44882 |

Source: Authors’ illustrations based on STATA software.
