# Supplementary material for: The cost of aging: Economic growth perspectives for Europe
Source: PLoS One. 2023 Jun 23;18(6):e0287207. doi: 10.1371/journal.pone.0287207 (PMC10289460; doi:10.1371/journal.pone.0287207)
Supplement: S6 Appendix — (DOCX) [file pone.0287207.s006.docx]

**S6 Appendix. VAR estimation**

|  | **Lags** | **PGDP** | | **EPOP** | |
| --- | --- | --- | --- | --- | --- |
|  |  | PGDP | EPOP | PGDP | EPOP |
| **Luxembourg** | 1 | 0.250102* | -7.5672 | -0.00413** | 2.005579*** |
|  | 2 | -0.0127 | 15.9819 | 0.003101* | -1.21536*** |
|  | 3 | 0.0854 | -8.9747 | 0.0010 | 0.1952 |
|  | **Lags** | **PGDP** | | **DDEPOP** | |
|  |  | PGDP | DDEPOP | PGDP | DDEPOP |
| **Austria** | 1 | 0.1566 | 1.6177 | -0.0045 | 0.5686272*** |
|  | 2 | 0.0068 | -2.6631 | 0.0056 | 0.1104 |
|  | 3 | 0.3490805** | 6.5226 | -0.006863** | -0.2326898* |
| **Belgium** | 1 | -0.0654 | -0.0949 | -0.0027 | 0.461894*** |
|  | 2 | 0.255646* | 4.5223 | 0.0016 | 0.0636 |
|  | 3 | 0.251313* | 8.3056 | -0.0054 | -0.1750 |
| **Denmark** | 1 | 0.1905 | -2.6606 | -0.0014 | 0.601373*** |
|  | 2 | -0.0574 | 0.9017 | 0.005377** | -0.0204 |
|  | 3 | 0.0983 | -5.9248 | -0.00523** | -0.0881 |
| **Finland** | 1 | 0.534479*** | -3.9367 | 0.0001 | 0.590451*** |
|  | 2 | -0.31084** | -10.5361 | 0.0017 | 0.1491 |
|  | 3 | 0.1875 | 14.1620 | -0.0006 | -0.1883 |
| **France** | 1 | -0.0146 | 1.5714 | -0.0023 | 0.809937*** |
|  | 2 | 0.2251 | 7.6918 | 0.006564* | -0.0361 |
|  | 3 | 0.3163 | 0.8893 | -0.00642* | -0.2508 |
| **Greece** | 1 | 0.267129** | 1.2462 | 0.0001 | -0.0104 |
|  | 2 | 0.1041 | 6.8553 | -0.0041 | -0.0105 |
|  | 3 | 0.243886* | 23.38649*** | 0.0028 | 0.0612 |
| **Italy** | 1 | 0.0923 | 3.0981 | -0.0003 | 0.782327*** |
|  | 2 | 0.1005 | 8.9111 | 0.0028 | 0.0085 |
|  | 3 | 0.33486** | -1.2566 | -0.0033 | -0.32524*** |
| **Netherlands** | 1 | 0.345871** | -11.2106 | -0.00075 | 0.538652*** |
|  | 2 | -0.13451 | 13.21251 | 0.000326 | 0.106142 |
|  | 3 | 0.199436 | -12.3257 | -0.00024 | -0.09665 |
| **Norway** | 1 | 0.522066*** | 0.3025 | 0.0020 | 0.495617*** |
|  | 2 | 0.0804 | -12.2963 | -0.0038 | 0.0849 |
|  | 3 | -0.0603 | 3.8121 | -0.0002 | 0.0156 |
| **Portugal** | 1 | 0.307318** | 13.7126 | -0.0001 | 0.465337*** |
|  | 2 | -0.0142 | 29.1760 | -0.0001 | 0.1078 |
|  | 3 | 0.300053** | -0.5507 | -0.00107 | -0.1710 |
| **Spain** | 1 | 0.2376 | -7.6690 | -0.0002 | 0.4752 |
|  | 2 | 0.1075 | 9.2827 | -0.0061 | 0.1565 |
|  | 3 | 0.2187 | 8.2140 | 0.0045 | -0.1887 |
| **Sweden** | 1 | 0.240435* | 5.5685 | 0.005186*** | 0.289324** |
|  | 2 | -0.2264 | 3.7369 | 0.000194 | 0.143967 |
|  | 3 | 0.0463 | 13.8818 | -0.00188 | 0.06514 |
| **Turkey** | 1 | -0.08471 | 66.42093 | 0.000181 | 0.613412*** |
|  | 2 | -0.05479 | 19.85794 | 7.79E-05 | 0.154121 |
|  | 3 | -0.06903 | -70.7684* | 9.55E-05 | -0.0461 |
| **United Kingdom** | 1 | 0.096914 | 3.922954 | -0.00205 | 0.32998** |
|  | 2 | 0.071376 | 26.16954*** | -0.00096 | 0.092204 |
|  | 3 | 0.000131 | -14.394 | 0.00306 | -0.08649 |

Source: Authors’ illustrations based on STATA software.
