# Supplementary material for: The cost of aging: Economic growth perspectives for Europe
Source: PLoS One. 2023 Jun 23;18(6):e0287207. doi: 10.1371/journal.pone.0287207 (PMC10289460; doi:10.1371/journal.pone.0287207)
Supplement: Attachment — Submitted filename: Response to the Reviewers.docx [file pone.0287207.s007.docx]

**Point by point response to editor and reviewers**

Dear editor and the reviewers,

We would like to express our profound appreciation to the editor and the reviewers for the valuable comments and suggestions made on our manuscript which were very helpful in revising and improving it.

Please note that the line numbers referred in this document is aligned with the revised manuscript which has track changes.

| **Comments of the Reviewer** | **Comments of the Authors** |
| --- | --- |
| 1. If the authors have adequately addressed your comments raised in a previous round of review and you feel that this manuscript is now acceptable for publication, you may indicate that here to bypass the “Comments to the Author” section, enter your conflict-of-interest statement in the “Confidential to Editor” section, and submit your "Accept" recommendation.  Reviewer #1: (No Response)  Reviewer #2: (No Response) | Well noted your comment with thanks. |
| 2. Is the manuscript technically sound, and do the data support the conclusions?  The manuscript must describe a technically sound piece of scientific research with data that supports the conclusions. Experiments must have been conducted rigorously, with appropriate controls, replication, and sample sizes. The conclusions must be drawn appropriately based on the data presented.  Reviewer #1: Partly  Reviewer #2: Yes | Well noted your comment with thanks. Comments have been incorporated in the revised version. |
| 3. Has the statistical analysis been performed appropriately and rigorously?  Reviewer #1: Yes  Reviewer #2: Yes | Duly noted! Thank you! Response well considered and the statistical analysis expanded. |
| 4. Have the authors made all data underlying the findings in their manuscript fully available?  The [PLOS Data policy](http://www.plosone.org/static/policies.action#sharing) requires authors to make all data underlying the findings described in their manuscript fully available without restriction, with rare exception (please refer to the Data Availability Statement in the manuscript PDF file). The data should be provided as part of the manuscript or its supporting information, or deposited to a public repository. For example, in addition to summary statistics, the data points behind means, medians and variance measures should be available. If there are restrictions on publicly sharing data—e.g. participant privacy or use of data from a third party—those must be specified.  Reviewer #1: Yes  Reviewer #2: Yes | Noted with thanks! Authors ensured all data underlying the findings are available in the manuscript. |
| 5. Is the manuscript presented in an intelligible fashion and written in standard English?  PLOS ONE does not copyedit accepted manuscripts, so the language in submitted articles must be clear, correct, and unambiguous. Any typographical or grammatical errors should be corrected at revision, so please note any specific errors here.  Reviewer #1: No  Reviewer #2: Yes | Comment well noted with thanks. Language has been improved. |
| **REVIEWER #1** | |
| **Comment 1** |  |
| The paper constitutes an attempt to analyse demographic and GDP figures spanning six decades from a number of (but not all) western, northern and southern European countries. (This includes a country located mainly in western Asia, no eastern, and only one central European country, involving half the current EU member states and a small number of non-EU countries.) The papers find that with a low probability of error:  (1) Changes in the changes of the population share of the elderly in two countries systematically preceded changes in the per capita GDP growth rate. (Incidentally, these two countries are one that exited the EU, and one that came close to exiting the EU).  (2) The opposite occurred in two Scandinavian EU member states. All in all, it finds evidence of heterogeneity (and perhaps there is more heterogeneity internally, at the subnational level). Yet, instead on following its own findings, instead of diversity, the paper sees closeness/oneness. I.e., countries that behave as “European member states” (top of p.3),  and operate as if the are part of a unit: “Europe has allocated funds… to manipulate the Europe based… plan” (bottom of p.3), “Europe have [has] proposed… policies” (p.5, bottom paragraph), “Europe has a very conservative strategy…” (p.6, bottom paragraph).  So, after two successive revisions, it is an unconvincing paper. | The comment is duly noted with many thanks!  According to the United Nations, there are 44 countries in Europe as of right now. 27 member states, most of which are in Europe, make up the supranational political and economic union known as the European Union (EU). Scandinavia, historically Scandia, part of northern Europe. In general, Scandinavia denotes Norway, Sweden, and Denmark.  The nations associated with the European Union and Scandinavian countries are, however, those found in Europe.  So, we changed it to word “European countries” in the cases you mentioned.  This has been incorporated in the revised manuscript from line numbers 45-46  “…European countries are currently experiencing an ageing population due to rising longevity and falling fertility rates….”  This study's main contribution is to investigate the relationship between economic growth and population aging in European countries. This study uses the most recent data gathered over a long |
| **Comment 2** |  |
| However technically sound its bivariate analysis may be, the discussion of the results supplies no explanation about the following:  (A) Why in some cases (i.e., in some countries) demographic changes that occurred 2-3 years ago forecast/forerun (they do not necessarily cause) per capita GDP changes today, whereas in other cases this does not happen? (NB: In the latter countries the impact is statistically insignificant.) Was it due to governmental supply- or demand-side policies or due to external shocks (crises) or something else? (B) Why in some other cases, per capita GDP changes observed 2-3 years ago forecast/forerun demographic changes today (demographic changes which to some or considerable extent are based on procreation decisions and medical advances of previous decades), and in other cases this practically does not happen? (C) Why in most of the cases (i.e., cases of original EU member states, of recent EU member states, as well as of non-EU member states) neither of the two happens? At what threshold or for what reason the societies/economies considered behave (act or react) so differently? Interestingly, without identifying what each of the aforesaid economies or societies did differently, the paper’s conclusions-and-policy-implications chapter recommends similar things for all.  E.g., in one case, to do what Greece and Italy do, when in fact Greece is placed in category (A) and Italy is placed in category (C). | Thanks for the detailed comment and suggestions.  The two factors of aging population and economic growth are related for a variety of reasons. When those factors are relatively weak in a country, there is less of an impact and linkage between these two variables.  Further explanation provided in the revised manuscript from line 398-413  “…. There are opportunities for increasing output per capita as a result of the demographic "dividend" for two reasons. The ratio of "producers" to "consumers" increases as the proportion of people in working age to the overall population rises. This has two effects. This undoubtedly has a positive impact on the rise in output per person. Second, "behavioural effects" on the growth of output per capita may also be present [48]. In countries such as Greece, UK etc., there is a substantial working elderly population. In any country, factors such as fertility, mortality, migration, and life expectancy have an impact on the ageing process [43]. There is no significant impact or change in the rate of economic growth when these factors occur in a slight and typical way. In contrast, an increase in the elderly population reduces economic growth because it dilutes capital [48]. The main policy consequences show that reducing government support for health care plans, pension plans and investing pension funds in high yield mutual and bond markets can increase the return on investments made by the government in the elderly population. Government supply- or demand-side policies, as well as external shocks prevent the implementation of such policies for the elderly population, which lessens the impact of the economy on this population. There is no significant relationship between these two variables when these factors are not as strong as it was….” |
| **Comment 3** |  |
| To the extent the analysis is based on statistical significance levels, the authors’ choice to band together significant and insignificant results as if they are equivalent (by reason that they collectively account for over 50% of the sample) is not persuasive. | Well noted and thanks for the valuable comment.  The World Bank's online database was where most secondary data were found. However, because of data availability, only 15 countries' worth of data were gathered for our study, which focused on the years between 1961 and 2021. If researchers examine the significant level for all European countries, this methodology is crucial. However, because we have 60 years' worth of data, it is challenging to find data for some countries. |
| **Comment 4** |  |
| The authors’ position in p.7 (3rd paragraph) that a decline in productivity observed in Finland or Sweden (the suggestion is for one country, but which of the two?) during the pandemic, was due to people’s ageing, is not well substantiated. To begging with, has the impact of the pandemic been econometrically isolated? Furthermore, the reference redirects to a host of internet archives, which is not very helpful. | Thank you very much for the comment and this is well noted. Revised manuscript has addressed the comment in line numbers 150-151.  “…. However, concerning the impact of aged population, Finland has reported the highest impact on their GDP growth. During 2019-2021….”  The World Bank's online database was used to obtain the majority of secondary data. However, the time frame of our study was from 1961 to 2021. Furthermore, the variables that change during a pandemic that lasts two or three years are not taken into account separately when using a large data set, such as 60 cases. This has been incorporated in line number 499-501 in revised manuscript as limitation.  “…In addition, when using a large data set such as 60 cases, the change in variables associated with a pandemic of two or three years is not taken into consideration separately….” |
| **Comment 5** |  |
| Crucial terms, such as PGDP, should be explained in the first encounter in the main text -not in subsequent paragraphs. | Thank you. Your comment is well received.  PGDP denotes the Per Capita Gross Domestic Product. When it is first mentioned in the study, it is described in line number 92.  “…the wavelet technique hasn’t been used in previous research to analyse the relationship between Per Capita Gross Domestic Product (PGDP) and the elderly population….” |
| **Comment 6** |  |
| Also, the phrase at the bottom of p.8, which relates that the parameters in Equations (1) and (2) are identical, but the regression parameters have an individual dimension, is confusing and has to be reworked. | Thank you very much for the comment and this is well noted.  Parameters in the equation (2) have changed according to the comment and it have been incorporated in line number 180.  ${EPOP}_{t}=Z+ \sum_{k=1}^{p} \gamma_{i} {EPOP}_{t-k}\sum_{k=0}^{p} \sigma_{i} {PGDP}_{t-k}+\varepsilon_{t}\ldots\ldots\ldots.(2)$ |
| **Comment 7** |  |
| The reason for discussing British industries, but not the industries of other countries, in the middle of page 11, remains elusive. | The comment is duly noted with many thanks!  Traditional industries were also significantly downgraded in other countries; as a result, line numbers 220-222 have been incorporated to the correction manuscript.  “….In Europe, the 1973 oil crisis, stagflation, the decline of traditional industries, inefficient production, and high inflation contributed to wage disputes [33]….” |
| **Comment 8** |  |
| The evidence (in the conclusions, p.24, 4th line) that pension care, government subsidies for the elderly and the overall policy developed for elderly care are strategised from the grassroots levels, is unclear. | Thank you very much for the comment and this is well noted.  The authors decided to eliminate the sentence after careful consideration. The revised manuscript contains a change in line numbers 447-449. |
| **Comment 9** |  |
| It is also unclear which is the “only one factor” that related to employment and retirement policies (in p.25, 6th line from bottom). | Thank you for the comment.  These changes have been incorporated in line number 489-491 in revised manuscript.  “…Employment and retirement policies are the result of many factors, and only one related to ageing can help to make public expenditure sustainable, and can contribute to healthy ageing [45]…” |
| **Comment 10** |  |
| The language needs to be further improved (for instance, the “United Nations” in p.5; “the Finland” in p.7, “15 Augest 2022” in p.27;the use of “Therefore” in p.5.) | Many thanks for the comment.  Change is visible in line numbers 102, 150-151, 543, 549, 109-110 in the revised manuscript  “…. Several leading institutions, including the United Nations, International Monetary Fund…”  “…. the impact of aged population, Finland has reported the highest impact on their GDP growth…”  “…cited 2022 15 Augest…”  “…. The estimated ageing population growth for Europe has exceeded 20% in the past three years and is expected to further increase….” |
| **Comment 11** |  |
| The text might flow better if Equation (3) were placed after the paragraph (or after the first lines of the paragraph) commencing with “Afterwards” in p.9. | The comment is duly noted with many thanks!  These changes have been incorporated in line number 188-189 in revised manuscript  “…. Afterwards, the wavelet coherence approach was used to visually supplement the results obtained from the panel Granger causality test….”  $\psi^{a,b}\left( x \right)= \left\vert a \right\vert^{-\frac{1}{2}}\psi\left( \frac{x-b}{a} \right)\ldots\ldots\ldots\ldots(3)$ |
| **Comment 12** |  |
| To the extent “western civilization” is not limited to the countries considered, and one of the countries considered does not fall into the category, the argument of p.3 (2nd paragraph) is not a very persuasive one. | Thank you very much for the comment and this is well noted.  After careful thought, authors decided to withdraw the sentence. Change is visible in line number 53 in the revised manuscript. |
| **Comment 13** |  |
| Which are the “others” (other variables) in p.24 (end of 1st paragraph)? | Noted with thanks your comment.  The elderly population and economic growth are the two endogenous variables in this study, so in wavelet coherence technique we conclude the dominant variable is the elderly population and other variable is economic growth. Revised manuscript has addressed the comment and changes in line numbers 452-454  “…Nevertheless, the additional insights from the wavelet coherence approach show that the dominant variable (elderly population) negatively leads the other variable (economic growth) during most periods….” |
| **REVIEWER 2** | |
| **Comment 1** |  |
| Concerning the authors’ response on my comment (3) in my previous report, I would kindly ask from authors to do the same with the subcommand “trend”. Please discuss the results involving this subcommand. | Thank you for the comment.  We used two commands called “dfuller” and “pperron” to see the stationarity of variables. The results are shown in Tables 2 and 3. But considering your comment, we run the “dfuller trend” command in STATA and it was seen that this is the same as the results obtained in table 2. The result table is attached Appendix S3. As a consequence, the other appendix numbers have been updated in the revised manuscript. |
| **Comment 2** |  |
| Please provide a short discussion (1-2 sentences) concerning the association between Granger-causality test (Table 4) and VAR results (S5 in Appendix). | Many thanks for the comment.  Here, we focus specifically on the Granger causality test. Under the previous response to the reviewer document, the VAR model results were mentioned. However, in response to your request, we have incorporated association between Granger-causality test and VAR model in line numbers 308-313.  “…. VAR models can be used to investigate relationships between the variables because these describe the joint generation process of several variables over time. One type of relationship between time series is Granger causality. Granger causality is an effective method for describing the interdependence of time series in reduced-form VAR. Granger causality test statistics are frequently used to determine whether lagged values of one variable contribute to the prediction of another variable [39]…..” |
| Additional Editor Comments: | |
| Please focus on the referees' concerns and comments and address them in a clear and detailed fashion. The empirical analysis part merits clear improvements in its explanation and methods. | Highly appreciate your comment and well noted.  We concentrated on the referees' concerns and comments, responding to them in a clear and detailed manner, and improving our study. |

Most recent revisions

| **Comments of the Reviewer** | **Comments of the Authors** |
| --- | --- |
| 5. Is the manuscript presented in an intelligible fashion and written in standard English?  PLOS ONE does not copyedit accepted manuscripts, so the language in submitted articles must be clear, correct, and unambiguous. Any typographical or grammatical errors should be corrected at revision, so please note any specific errors here.  Reviewer #1: No  Reviewer #2: Yes | Comment well noted with thanks. Language has been improved, with typographical or grammatical errors corrected. |
| **REVIEWER #1** | |
| **Comment 1** |  |
| The paper constitutes an attempt to analyse demographic and GDP figures spanning six decades from a number of (but not all) western, northern and southern European countries. (This includes a country located mainly in western Asia, no eastern, and only one central European country, involving half the current EU member states and a small number of non-EU countries.) The papers find that with a low probability of error:  (1) Changes in the changes of the population share of the elderly in two countries systematically preceded changes in the per capita GDP growth rate. (Incidentally, these two countries are one that exited the EU, and one that came close to exiting the EU).  (2) The opposite occurred in two Scandinavian EU member states. All in all, it finds evidence of heterogeneity (and perhaps there is more heterogeneity internally, at the subnational level). Yet, instead on following its own findings, instead of diversity, the paper sees closeness/oneness. I.e., countries that behave as “European member states” (top of p.3),  and operate as if the are part of a unit: “Europe has allocated funds… to manipulate the Europe based… plan” (bottom of p.3), “Europe have [has] proposed… policies” (p.5, bottom paragraph), “Europe has a very conservative strategy…” (p.6, bottom paragraph).  So, after two successive revisions, it is an unconvincing paper. | The sections highlighted have been further revised, pages 4 and 7. |
| **Comment 2** |  |
| However technically sound its bivariate analysis may be, the discussion of the results supplies no explanation about the following:  (A) Why in some cases (i.e., in some countries) demographic changes that occurred 2-3 years ago forecast/forerun (they do not necessarily cause) per capita GDP changes today, whereas in other cases this does not happen? (NB: In the latter countries the impact is statistically insignificant.) Was it due to governmental supply- or demand-side policies or due to external shocks (crises) or something else? (B) Why in some other cases, per capita GDP changes observed 2-3 years ago forecast/forerun demographic changes today (demographic changes which to some or considerable extent are based on procreation decisions and medical advances of previous decades), and in other cases this practically does not happen? (C) Why in most of the cases (i.e., cases of original EU member states, of recent EU member states, as well as of non-EU member states) neither of the two happens? At what threshold or for what reason the societies/economies considered behave (act or react) so differently? Interestingly, without identifying what each of the aforesaid economies or societies did differently, the paper’s conclusions-and-policy-implications chapter recommends similar things for all.  E.g., in one case, to do what Greece and Italy do, when in fact Greece is placed in category (A) and Italy is placed in category (C). | A sentence was added to address the concern on “recommends similar things for all”, page 28. |
